# Functional Genomic Analyses of Two Morphologically Distinct Classes of *Drosophila* Sensory Neurons: Post-Mitotic Roles of Transcription Factors in Dendritic Patterning

**DOI:** 10.1371/journal.pone.0072434

**Published:** 2013-08-15

**Authors:** Eswar Prasad R. Iyer, Srividya Chandramouli Iyer, Luis Sullivan, Dennis Wang, Ramakrishna Meduri, Lacey L. Graybeal, Daniel N. Cox

**Affiliations:** School of Systems Biology, Krasnow Institute for Advanced Study, George Mason University, Fairfax, Virginia, United States of America; Columbia University, United States of America

## Abstract

**Background:**

Neurons are one of the most structurally and functionally diverse cell types found in nature, owing in large part to their unique class specific dendritic architectures. Dendrites, being highly specialized in receiving and processing neuronal signals, play a key role in the formation of functional neural circuits. Hence, in order to understand the emergence and assembly of a complex nervous system, it is critical to understand the molecular mechanisms that direct class specific dendritogenesis.

**Methodology/Principal Findings:**

We have used the *Drosophila* dendritic arborization (da) neurons to gain systems-level insight into dendritogenesis by a comparative study of the morphologically distinct Class-I (C-I) and Class-IV (C-IV) da neurons. We have used a combination of cell-type specific transcriptional expression profiling coupled to a targeted and systematic *in vivo* RNAi functional validation screen. Our comparative transcriptomic analyses have revealed a large number of differentially enriched/depleted gene-sets between C-I and C-IV neurons, including a broad range of molecular factors and biological processes such as proteolytic and metabolic pathways. Further, using this data, we have identified and validated the role of 37 transcription factors in regulating class specific dendrite development using *in vivo* class-specific RNAi knockdowns followed by rigorous and quantitative neurometric analysis.

**Conclusions/Significance:**

This study reports the first global gene-expression profiles from purified *Drosophila* C-I and C-IV da neurons. We also report the first large-scale semi-automated reconstruction of over 4,900 da neurons, which were used to quantitatively validate the RNAi screen phenotypes. Overall, these analyses shed global and unbiased novel insights into the molecular differences that underlie the morphological diversity of distinct neuronal cell-types. Furthermore, our class-specific gene expression datasets should prove a valuable community resource in guiding further investigations designed to explore the molecular mechanisms underlying class specific neuronal patterning.

## Introduction

A complex nervous system consists of a vast number of neuronal classes, each displaying distinctive dendritic architecture. Dendritic branching pattern represents a hallmark of each neuronal type, and plays a functional role in signal-processing, neuronal function and circuit assembly [1]. Moreover, in humans, defects in dendritic development are among the strongest neuroanatomical correlates to neurological and neuro-developmental disorders including Down, Fragile X, and Rett syndromes as well as Autism [2], [3].

*Drosophila* dendritic arborization (da) sensory neurons have emerged as a powerful system to investigate class-specific dendritogenesis due to their distinct and well-characterized dendritic morphology (reviewed in [4]–[6]). The da neurons consist of 4 distinct morphological and functional classes (C-I-IV) of sensory neurons that have varying degrees of dendritic complexity [7]. Among da neurons, the class I (C-I) and class IV (C-IV) neurons represent examples of two extremes of dendritic complexity, where C-I neurons exhibit selective innervations of dendritic territories and occupy relatively small receptive fields, whereas C-IV neurons exhibit an elaborate space-filling network of dendrites that completely and non-redundantly tile the larval body wall [7].

The acquisition and maintenance of class-specific dendritic arbors is regulated by complex genetic and molecular programs involving both intrinsic factors and extrinsic cues [2]–[4]. While many genes and candidate-loci involved in the specification or maintenance dendrite morphology have been identified using forward genetic, gain-of-function and RNAi screens [8]–[14], we remain far from having a coherent mechanistic understanding of the processes governing class-specific dendrite development. Further, RNAi screens, without being guided by cell-type specific transcriptomic information, have frequently been observed to result in high false positive rates and ambiguous results [15]. In addition, many genes that contribute to complex morphogenesis programs may function in a range of developmental processes and are thereby expected to exhibit pleiotropy which can result in a failure to identify such morphogenesis genes in standard genetic screens [16]. In contrast, a reverse genetics-based functional genomics approach has the potential of presenting a more comprehensive, unbiased investigation of the genetic and regulatory programs operating at a class-specific level to drive dendritic arborization diversity by circumventing impediments introduced by genetic pleiotropy.

To this end, here we report the first global gene-expression profiles from purified *Drosophila* class I and IV da neurons using methods and protocols for neuronal cell-type specific isolation and gene expression profiling developed previously in our lab [17], [18]. From this dataset, we have identified gene-sets that are enriched uniquely within these two neuronal subtypes, and also those that are enriched commonly. Further, using this data, we have identified 40 differentially expressed transcription factors (TFs) and functionally validated the role of 37 TFs in regulating class specific dendrite development using RNAi knockdown followed by quantitative neurometric analysis. This study also reports the first large-scale neurometric analyses of over 4,900 reconstructed da neurons used to quantitatively validate the RNAi screen phenotypes. Overall, these analyses shed novel light on the molecular differences that underlie neuronal type-specific dendritic arborization. Furthermore, the class-specific gene expression profiles will prove to be a valuable resource in guiding further investigations designed to explore the cellular and molecular mechanisms underlying class-specific dendrite development.

## Results

### Microarray gene expression profiling from enriched C-I and C-IV da neuron populations

**Figure 1:**
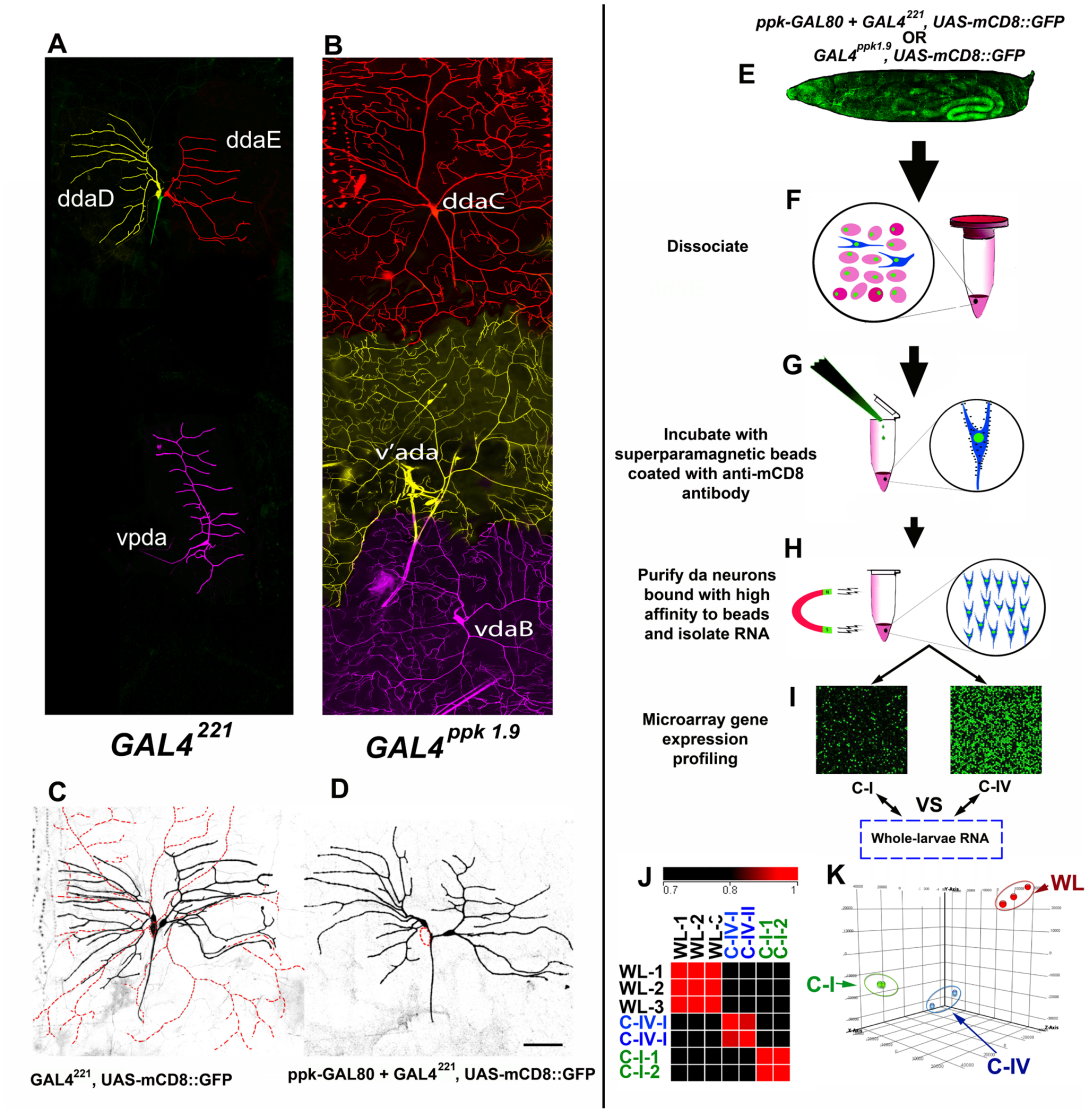
Microarray analysis of two morphologically distinct classes of *Drosophila* sensory neurons. To access differences in gene expression, RNA from enriched C-I and C-IV neuronal populations were compared with that of whole larvae in microarray experiments. Live confocal images of neuronal populations labeled by (**A**) *GAL4^221^* (C-I) and (**B**) *GAL4^ppk1.9^* (C- IV) visualized by the trans-membrane fusion construct mCD8::GFP. Neurons have been pseudo-colored to distinguish individual subtypes and dendritic territories. (**C**) *GAL4^221^* strongly labels C-I da neurons along with weakly labelling of C-IV neurons in the background (dotted red trace). (**D**) *GAL80* driven by a *ppk* promoter was combined in the background of *GAL4^221^(ppk-GAL80; GAL4^221^*) that results in highly class I specific *GAL4* expression. (**E**) A representative whole larval image of *GAL4^ppk1.9^* driving the expression of *UAS-mCD8::GFP* (gut is auto-fluorescent). (**F-J**) Strategy of class-specific neuronal isolation. Larvae expressing mCD8::GFP under the control of either *ppk-GAL80; GAL4^221^* or *GAL4^ppk1.9^* (**F**) were dissociated (**G**) filtered and incubated with superparamagnetic beads coated with anti-mCD8 antibody (**H**). The C-I/C-IV neurons bound to the magnetic beads were purified using a strong magnet (**I**), washed several time and used to perform microarray gene expression profiling (**J**). An identical region from the C-I and C-IV microarray are represented to show their dramatic qualitative differences (**J**). The microarray replicates were highly correlated, as represented in the correlation map (**K**). Principle component analysis revealed the three microarray samples from C-I, C-IV and whole larval lysate cluster into three distinct and well-defined clusters (**L**).

In order to obtain an unbiased and global profile of the putative mechanisms regulating class-specific dendritic arborization, we performed comparative microarray analyses on highly enriched cell populations from C-I and C-IV da sensory neurons of the *Drosophila* peripheral nervous system (Fig. 1). These two subclasses of da neuron were chosen due to their dramatically different degrees of dendritic complexity and as a means to identify transcriptional programs underlying differential dendritogenesis. To achieve this, we employed a rapid magnetic bead based cell sorting strategy in combination with the *GAL4-UAS* system, which we have previously demonstrated to yield highly enriched populations of individual neuronal subclasses [17]–[20]. *GAL4^221^* and *GAL4^ppk1.9^* were individually recombined with the *UAS-mCD8::GFP* reporter in order to genetically label C-I and C-IV neurons, respectively. These *GAL4* drivers were selected based on their strong and well characterized expression patterns within these specific da neuron subclasses [10], [21], [22]. *GAL4^ppk1.9^* has a highly specific C-IV expression, labelling all three C-IV neurons (ddaC, v’ada, vdaB) (Fig. 1B). G*AL4^221^* drives expression in all three C-I neurons (ddaD, ddaE and vpda) (Fig. 1A), but also labels C-IV da neurons at a low level [23] (Fig. 1C). In order to restrict the G*AL4^221^* expression to class I only, we subtracted C-IV expression by co-expressing *GAL80* driven by a *ppk* promoter [24] thereby resulting in a highly specific C-I driver (Fig. 1D).

Using these highly specific C-I and IV *GAL4* drivers, we isolated class-specific da neuron populations from age-matched third instar larvae via magnetic bead sorting and conducted comparative microarray gene expression profiling (Fig. 1E-I). To assess the enrichment of da neurons, we extracted total RNA and conducted qRT-PCR analyses using PNS and neuronal gene markers including *futsch* and *elav* which revealed these markers were highly enriched (data not shown) [17]. Moreover, we visually inspect each cell isolation for purity by verifying that isolated neurons are GFP positive and that there is an absence of contaminating GFP negative cells. To independently assess the purity of the cell isolations, we expressed a *UAS-cut* transgene in both C-I and C-IV da neurons and compared the mRNA expression levels relative to controls lacking the *UAS-cut* transgene via qRT-PCR. For these analyses, control or *UAS-cut* overexpressing C-I or C-IV da neurons were isolated via magnetic bead sorting, total RNA was extracted, and *cut* expression levels were analyzed between genetic backgrounds. These analyses revealed highly significant upregulation of *cut* in C-I (14±4 fold) and C-IV (4.4±1.2 fold) relative to control neurons which is indicative of the purity of the class-specific cell isolations (Fig. S1). Moreover, the fold difference in *cut* expression levels is consistent with the previously reported differential expression levels of Cut in da neuron subclasses [22].

Total RNA derived from whole larvae homogenate (control) and highly enriched populations of C-I and C-IV da neurons were then used to conduct whole genome microarray expression profiling. For these studies, single-channel Cy3-labeled amplified cDNAs were used as probes against the Agilent whole genome *Drosophila melanogaster* oligo microarray (4×44K) platform in biological replicates producing strong signal intensities and high reproducibility with an average inter-replicate Pearson’s correlation coefficient of 0.97 (Fig. 1K). Stringent data filtering parameters were employed to remove potential false-positive data-points and retain only the high-confidence expression values. Briefly, arrays were quantile normalized and low-quality spot expression values were filtered out based on expression flags followed by one-way ANOVA and Tukey's Honestly Significant Difference (HSD) test with a corrected p-value cut-off ≤0.01. Principle Component Analysis (PCA) analysis was conducted on the array data, which revealed all arrays to segregate into three well-defined and distinct clusters (Fig. 1L).

### Comparative transcriptomic analyses of C-I and C-IV *Drosophila* da neurons

**Figure 2:**
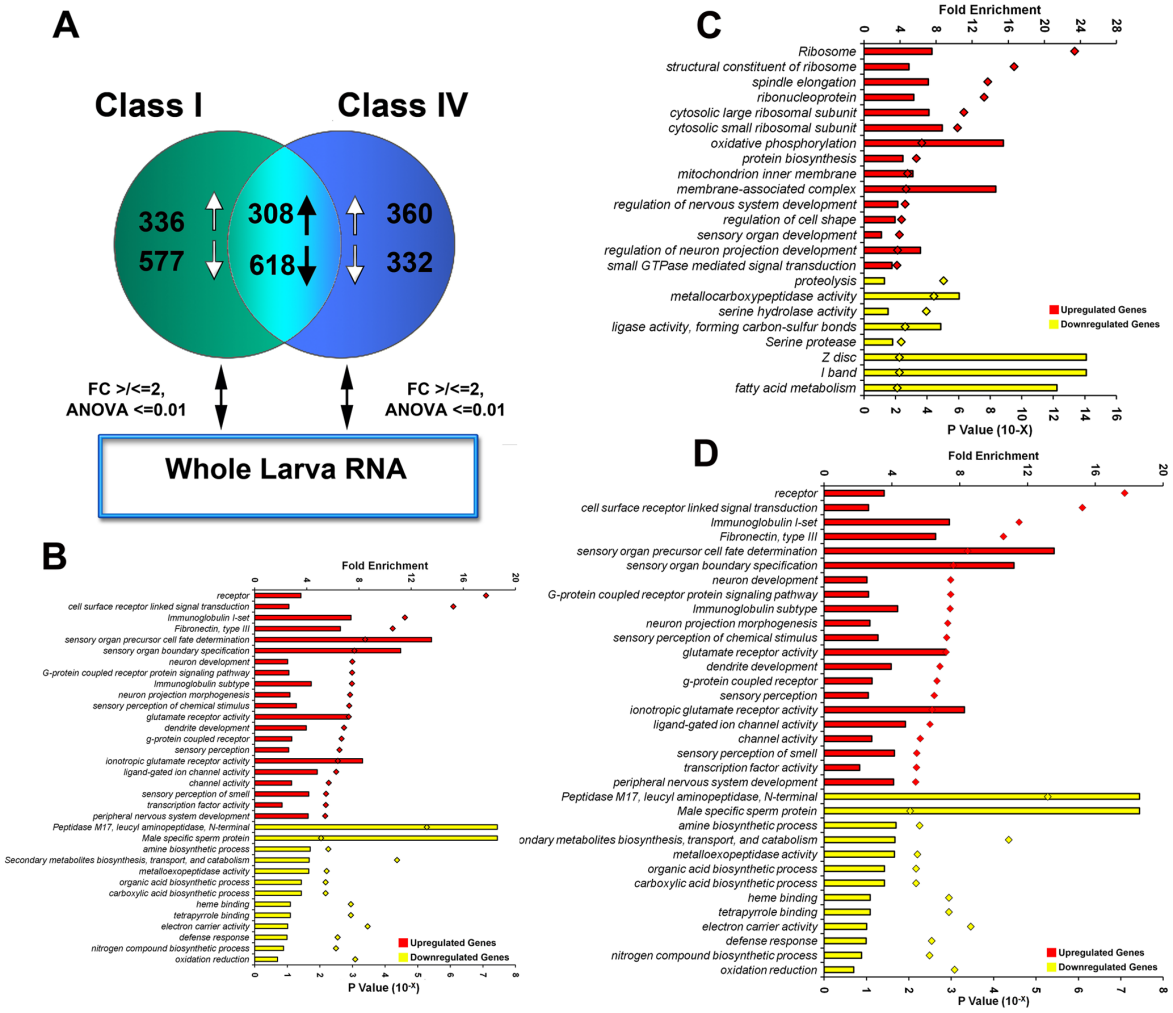
Functional characterization of differentially expressed gene-sets in C-I and C-IV neurons. (**A**) Venn diagram representing the number of genes differentially upregulated/downregulated either uniquely or commonly in C-I and C-IV da neurons with respect to whole larvae controls. (**B-D**) Analysis of gene ontology (GO) categories for genes that are enriched/depleted in C-I and C-IV neurons. The graph represents GO categories that are significantly over-represented (P≤0.01) in the population of differentially expressed genes that are uniquely regulated in C-I neurons (**B**), uniquely regulated in C-IV neurons (**C**) or commonly regulated in both C-I and C-IV neurons (**D**) when compared to whole larval controls. Bars indicate the fold enrichment (top X axis) of the genes belonging to a given GO term in the population of regulated genes in comparison to the total population of genes in the Agilent 4×44k array. Diamonds indicate the Modified Fisher’s Exact p-value (EASE score, bottom X axis) for each category.

To identify genes significantly enriched/depleted within each neuronal class, we compared the gene expression profiles of purified C-I and C-IV da neurons to wild-type whole larval lysate samples (Fig. 2A). Of the 13,028 unique genes represented in the 4×44 k array, a relatively small number of a few hundred genes were found to be differentially regulated in da neurons as compared to the whole larval controls. These genes have been grouped into three distinct categories as follows: (A) genes uniquely enriched/depleted in C-I neurons, (B) genes uniquely enriched/depleted in C-IV neurons and (C) genes that were commonly enriched/depleted between C-I and C-IV neurons (Fig. 2A).

Consistent with our class-specific da neuron isolation, we observed significant expression over background for many genes previously implicated in dendrite development and da neuron specification including the microtubule associated molecule *futsch* which was enriched by over 80-fold and 10-fold in C-IV and C-I neuron samples, respectively [25] and the gene *absent MD neurons and olfactory sensilla* (*amos*) which was enriched by 13.9- and 2.5-fold in fold in C-I and C-IV neurons, respectively [26]. In contrast, some genes previously implicated in da neuron development, while identified in our microarray studies, were not found to be statistically enriched when compared to the background whole larval control arrays, potentially due to the high/ubiquitous expression of these genes in several larval tissue types. For example, while the BTB/POZ domain containing transcription factor *abrupt* is expressed at detectable levels in our C-I/IV microarrays [27], [28], it was not found to be statistically enriched in C-I or C-IV da neurons when compared to whole larval homogenate, likely due to its high expression in larval central nervous system and the imaginal discs [29]. Moreover, while we observed detectable *abrupt* mRNA expression in C-IV neurons, RNAi knockdown of *abrupt* in these neurons produced no statistically significant dendritic defects consistent with previously published reports [27], [28] (data not shown).

To explore the biological categories of genes potentially underlying C-I and C-IV neuron dendrite development, we examined functionally related groups of genes significantly enriched within these neurons. The DAVID functional enrichment tool [30], [31] was used to provide an unbiased approach to this type of analysis, highlighting functional groups of genes that co-occur and are also statistically enriched in a given gene population. The Gene Ontology (GO) consortium provides detailed annotations of genes with respect to their known or putative molecular function, cellular localization and biological processes [32], which is used by DAVID to identify functional classes of genes that are statistically over-represented amongst the genes differentially expressed in either C-I or C-IV da neurons with respect to whole larval control (Tables S1-S3). The enrichment of functional gene classes was quantitatively measured in DAVID by well accepted statistical methods including, *χ* ^2^, Fisher’s exact test, binomial probability and hypergeometric distribution.

Comparative analyses revealed a total of 360 and 332 transcripts that were found to be up/downregulated, respectively, greater than 2-fold in C-IV neurons as compared to whole larvae controls (p≤0.01, ANOVA, Tukey HSD test) (Fig. 2A). As predicted, numerous genes involved in sensory organ development and regulation of the nervous system were found to be highly enriched within this population (Fig. 2C, Table S2). In addition, among the group of genes upregulated in C-IV neurons, there is significant enrichment of genes involved in protein biosynthesis, ribosomal and ribonucleoprotein complex, oxidative phosphorylation and membrane-associated complex were also present (Fig. 2C, Table S2). The population of genes downregulated in C-IV neurons were enriched for genes involved in protein degradation, proteolysis and fatty acid metabolism (Fig. 2C, Table S2). In addition, muscle tissue related gene ontologies such as “Z-disc” and “I-band” were significantly downregulated in C-IV neuron sample (>14 fold, p≤0.01) indicative of the enriched purity of the isolated neurons apart from other contaminating cell types (Fig. 2C, Table S2).

In contrast to C-IV neurons, several genes involved in the process of protein catabolism like serine hydrolyse and carboxypeptidases were significantly upregulated in C-I neurons with respect to whole larvae controls (fold change ≥ 2, p≤0.01, ANOVA, Tukey HSD test) (Fig. 2A, Table S1). In addition to these, genes associated with the GO term “fibronectin, type III-like fold” and “immunoglobulin” were also significantly upregulated in C-I neurons (Fig. 2A, Table S1). The population of genes downregulated in C-I neurons were enriched for genes involved in the c-Jun N-terminal Kinase (JNK) and Mitogen-Activated Protein Kinase (MAPK) cascades (Fig. 2A, Table S1). Moreover, ontologies like “actin filament-based process”, “muscle organ development”, respiratory system development” and “epithelium development” were likewise significantly down regulated in C-I neurons consistent with neuronal specificity of the cell isolation and cytoskeletal distributions in C-I neurons [34] (Fig. 2A, Table S1).

In order to understand pathways commonly regulated in both C-I and C-IV neurons, we analyzed genes that were commonly up/downregulated in these neurons. A total of 308 genes were upregulated and 618 genes downregulated commonly in C-I and IV neurons with respect to whole larvae controls (fold change ≥ 2, p≤0.01, ANOVA Tukey HSD test). The group of genes commonly upregulated were highly enriched for genes belonging to the category of cell-surface receptor linked signal transduction, immunoglobulin, ion channels, cytoskeletal regulators and transcription factors (Fig. 2D, Table S3). As expected, these sensory neurons were also highly enriched for genes previously implicated in dendrite development, neuron projection morphogenesis and sensory perception (Fig. 2D, Table S3). The population of genes commonly downregulated in C-I and C-IV neurons were enriched for genes involved in amine biosynthesis, secondary metabolite and organic acid biosynthetic pathways (Fig. 2D, Table S3. In addition, genes involved in non-neuronal biological processes like reproduction and immune response were also significantly downregulated (Fig. 2D, Table S3).

In order to further understand the molecular differences between C-I and C-IV neurons, we investigated gene-sets that were inversely regulated between these two classes. For these analyses, we identified ontologies that were enriched among genes that were statistically upregulated in one class of neuron, and were simultaneously downregulated in the other neuronal class. We identified 204 genes that were both upregulated in C-IVs (≥ 2 fold), and also downregulated in C-Is (≤ 2 fold) (Table S4). Similarly, a total of 91 genes were upregulated in C-I’s (≥ 2 fold) and also downregulated in C-IV’s (≤ 2 fold) (Table S4). Functional enrichment analysis was performed on these gene lists to identify key biological pathways that might be inversely regulated between these two morphological distinct neuron subtypes. Interestingly, several genes involved in the process of energy production in the mitochondria were one of the most inversely regulated pathways. For example, the GO terms “electron transfer” and “oxidative phosphorylation” were enriched by over 30-fold and 22-fold, respectively, among the genes that were upregulated in C-IVs and also downregulated in C-Is (Table S4). These included the mitochondrial associated genes *Cytochrome b* (*mt:Cyt-b*), *Cytochrome c oxidase subunit II* (*mt:CoII*), *Cytochrome c oxidase subunit III* (*mt:CoIII*) and the *mitochondrial ATPase subunit 8* (*mt:ATPase8*). This suggests that one of the key differences between C-I and C-IV might be in the area of energy production. Conversely, we analysed the list of genes positively regulated in C-I neurons but inversely regulated in C-IV neurons. Functional enrichment analysis of these 91 genes revealed that genes involved in catabolism were most enriched. For example, the GO term “Peptidase M14, carboxypeptidase A”, “metallopeptidase activity” and “proteolysis” were among the most significantly enriched. These included several genes with uncharacterized functions such as *CG14820*, *CG15253* and *CG18557* in addition to genes with well-characterized functions such as *serine protease 6* and *multi-drug resistance 50* (*Mdr50*) (Table S4). Collectively, these results suggest that proteolytic and metabolic pathways may be one of the core differentially regulated mechanisms underlying dendritic morphological diversity. Moreover, these comparative transcriptomic analyses suggest numerous testable hypotheses for future research into the molecular underpinnings of class specific dendrite arborization.

### Microarray functional validation via *in vivo* RNAi screen and quantitative neurometric analyses

**Figure 3:**
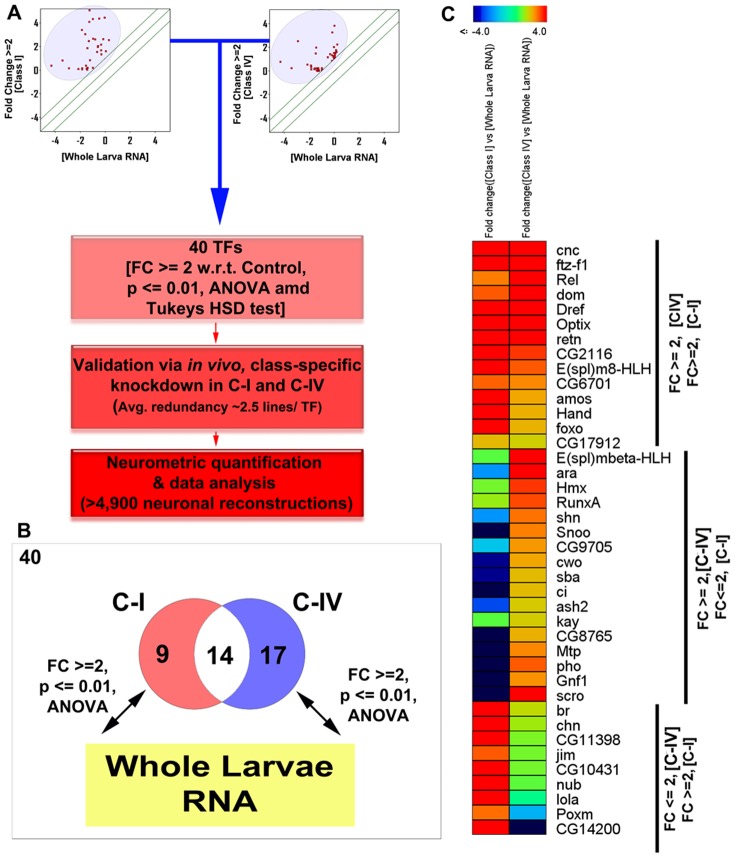
Identification of differentially enriched transcription factors in C-I and IV neurons. (**A**) A set of 40 TFs were found to be specifically enriched within C-I and C-IV da neurons in comparison to whole-larvae controls (p≤0.01, ANOVA and Tukeys HSD test), of which 9 were enriched uniquely within C-I neurons, 17 in C-IV neurons and 14 in both C-I and C-IV’s as represented in the Venn diagram (**B**). (**C**) The relative microarray fold-change expression values for the differentially expressed TFs in C-I and C-IV neurons, in comparison to whole larval control samples, are represented as hierarchically clustered heat map and the relative values are represented as a rainbow color scheme according to the designated scale (p≤0.01, ANOVA and Tukey’s HSD test).

Previous studies have implicated transcription factors (TFs) as key regulators of dendrite development including combinatorial TF activity as a mechanism to fine-tune dendritic branching architecture [4]–[6], [9], [22], [27], [28], [33]–[36]. While TFs clearly serve critical functional roles in regulating dendritogenesis, less is known regarding how differential TF expression may contribute to specification, maintenance or modulation of class-specific dendritic morphologies. To serve the dual purpose of validating our microarray data and identifying candidate molecules regulating class-specific dendrite development, we focused on the identification of TFs that were uniquely or commonly enriched in C-I and/or C-IV neurons compared to the whole-larval controls (excluding TFs with compromised expression in one or more arrays). In total, we identified a set of 40 TFs, of which 9 were uniquely enriched in C-I neurons, and 17 were uniquely enriched in C-IV neurons and 14 TFs were enriched in both C-I and C-IV neurons at a significantly higher levels than controls (fold change ≥ 2, p≤0.01 one-way ANOVA and Tukey's HSD test) (Fig. 3, Table S5).

Comparative microarray analyses revealed that almost twice the number of TFs are uniquely enriched in C-IV neurons (17) in comparison to C-I neurons (9) suggesting that the more complex C-IV neurons may require a larger number of TFs to regulate dendritic patterning relative to the simpler morphology exhibited by C-I neurons (Fig. 3A,B). While the total number of genes uniquely enriched within C-I and C-IV neurons are not vastly different (C-I; 336, C-IV; 360), C-IV neurons contain a larger enrichment of TFs than C-I (C-I; 2.6%, C-IV; 4.7%). To functionally validate the significance of these differential or common expression patterns, we performed a systematic *in vivo* RNAi screen for 37 of the 40 TFs in both C-I and C-IV da neurons under identical conditions using multiple gene-specific RNAi inverted repeat (IR) constructs (*UAS-IR*), given that for 3 of the 40 differentially expressed TFs multiple *UAS-IR* transgenes were not available (*sba*, *E(spl)mbeta-HLH*, and *CG14200*). To increase the RNAi knockdown efficiency, we enhanced the *GAL4* expression by rearing the progeny of the crosses and controls (*C-I/C-IV-GAL4* (X) *UAS-IR* or *OregonR*) at elevated temperatures (29°C) throughout development [38], [39] which did not, itself, produce any significant effects on da neuron dendrite morphology relative to room temperature (25°C).

**Figure 4:**
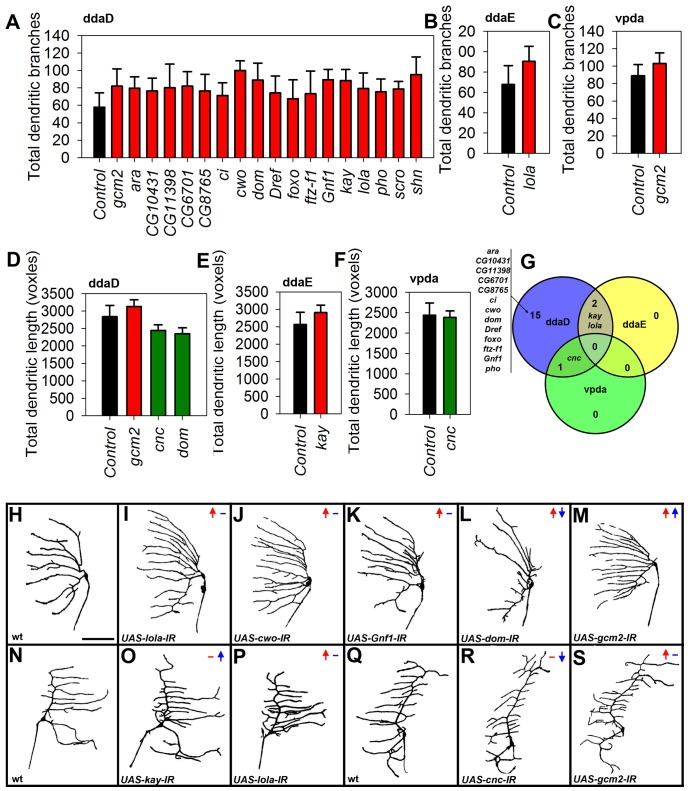
C-I TF screen quantitative phenotypic analyses. All TFs that resulted in C-I dendritic changes that were significantly different from controls in at least two independent RNAi lines have been shown as bar graphs where the N = 9 for each RNAi line tested per gene (p≤0.05, Student’s t-test). The bars have been color-coded to represent increase (red) or decrease (green) in values relative to controls (black). (**A-C)** Quantitative analyses of total dendritic branches is shown for ddaD (**A**), ddaE (**B**) and vpda (**C**). (**D-F**) Quantitative analyses of total dendritic length is shown for ddaD (**D**), ddaE (**E**) and vpda (**F**). (**G**) Venn diagram distribution of TF-induced phenotypes among the three C-I subtypes. (**H-S**) Representative images of selected RNAi-induced phenotypes observed in C-I da neurons. Live confocal images of wild-type (wt) and RNAi (*UAS-IR*) phenotypes in the three C-I subtypes labelled using *UAS-mCD8::GFP* driven by C-I *GAL4*. Compared to wild-type C-I neurons (**H, N, Q**), phenotypes of *UAS-lola-IR* (**I, P**), *UAS-cwo-IR* (**J**), *UAS-Gnf1-IR* (**K**), *UAS-dom-IR* (**L**), *UAS-kay-IR* (**O**) and *UAS-cnc-IR* (**R**) are shown. Panels (**M**) and (**S**) represent phenotypes of *UAS-gcm2-IR* which was used as a positive control. Phenotypic information for each image is represented by color-coded arrows, where red arrows represent dendritic branching and blue arrows represent total dendritic length. The direction of arrow represents increase (up), decrease (down) or no change (hyphen). Size bar corresponds to 50 microns.

**Figure 5:**
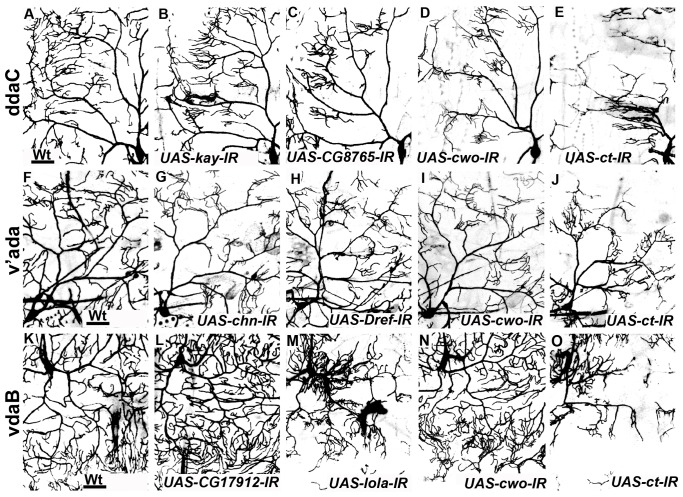
C-IV TF screen qualitative phenotypic analyses. Representative images of selected RNAi-induced phenotypes observed in C-IV da neurons. Live confocal images of wild-type and RNAi (*UAS-IR*) phenotypes in the three C-IV subtypes sub-labelled using *UAS-mCD8::GFP* driven by C-IV *GAL4*. Relative to wild type class IV neurons (**A, F, K)**, phenotypes of *UAS-kay-IR* (**B)**, *UAS-CG8765-IR* (**C**), *UAS-cwo-IR* (**D, I, N**), *UAS-chn-IR* (**G**), *UAS-Dref-IR* (**H**), *UAS-CG17912-IR* (**L)** and *UAS-lola-IR* (**M**) are shown. Panels **E, J, O** represent phenotypes of *UAS-cut-IR* which was used as a positive control. Size bar corresponds to 50 microns.

Numerous recent studies have demonstrated the utility of using *in vivo* RNAi knockdowns, which can not only effectively and reproducibly replicate mutant phenotypes, but also help circumvent the problem of genetic pleiotropy in *Drosophila* at embryonic, larval and adult stages [37], [40]–[43]. We too confirmed that knocking down known regulators of da neurons produced robust and quantifiable phenotypes by testing known regulators of dendrite development for inducing phenotypes. For example, knocking down *dar1* (data not shown) and *gcm2* (Fig. 4M,S) resulted in expected phenotypes in C-I neurons as has been previously reported in the literature [9], [44]. Similarly, knocking down the homeodomain TF *cut* in C-IV neurons using 3 independent *UAS-IR* constructs all resulted in strong and penetrant phenotypes in C-IVs as has been previously reported [22] (Fig. 5D,H,L). Conversely, *cut* RNAi in C-I neurons produced no phenotypes in all three *UAS-IR* lines consistent with previous findings [22] (data not shown). In addition, we verified the reproducibility of our RNAi phenotypes by testing multiple independent *UAS-IR* constructs against positive controls that were expected to affect dendritic morphology. For example, 4 independent *UAS-IR* constructs targeting the zinc-finger transcription factor *cubitus interruptus* (*ci*), involved in the Hedgehog signaling pathway, resulted in strong phenotypes in C-IV neurons with a significant reduction in dendritic branching (28±4.1%) and in total dendritic length (19±4.8%) when compared to wild-type controls which is consistent with previously published phenotypic data [45] (Fig. 5). Thus, RNAi-induced phenotypes were seen to result in consistent morphological defects that are reproducible in da neurons. Moreover, to determine if subtypes of morphologically similar C-I or C-IV da neurons exhibit molecular heterogeneity in regulating dendrite morphogenesis, we analyzed and compared the wild-type and TF knockdown phenotypes of each of the three C-I da neurons (ddaD, ddaE and vpda) as well as each of the three C-IV neurons (ddaC, v’ada and vdaB) using live confocal microscopy and rigorous neurometric quantification. Additionally, each TF was screened with a minimum of 2, and in some cases up to 5, independent *UAS-IR* transgenes (average redundancy ∼ 2.5 RNAi lines/TF).

**Figure 6:**
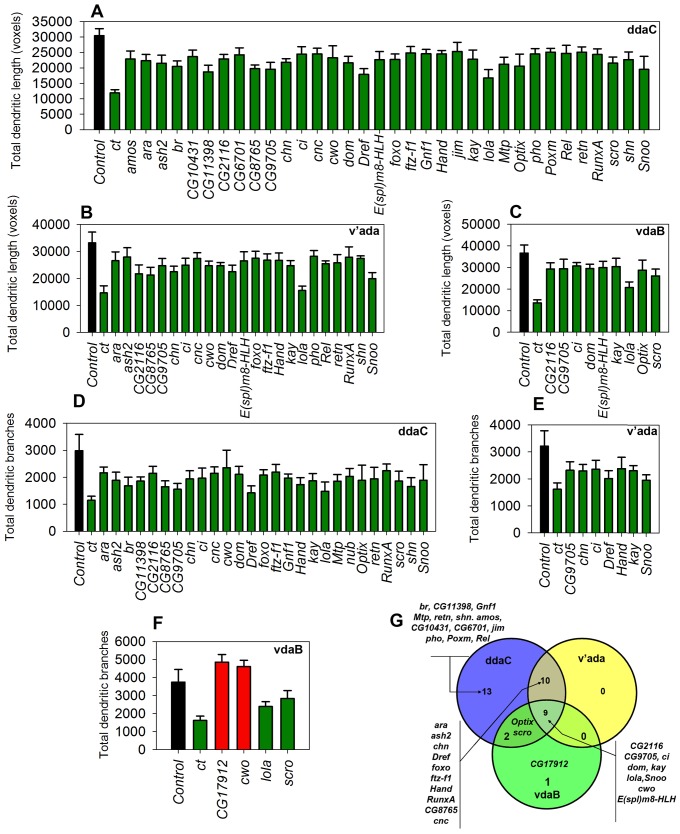
Neurometric quantitative analyses of C-IV TF phenotypes. (**A-F**) TFs that resulted in dendritic changes that were significantly different from controls in at least two independent RNAi lines have been shown as bar graphs where the average N = 7 for each RNAi line tested per gene (p≤0.05, Student’s t-test). The bars have been color-coded to represent increase (red) or decrease (green) in values relative to controls (black). (**A-C)** Quantitative analyses of total dendritic length is shown for ddaC (**A**), v’ada (**B**) and vdaB (**C**). (**D-F**) Quantitative analyses of total dendritic branches is shown for ddaC (**D**), v’ada (**E**) and vdaB (**F**). (**G**) Venn diagram distribution of TF-induced phenotypes among the three C-IV subtypes.

**Figure 7:**
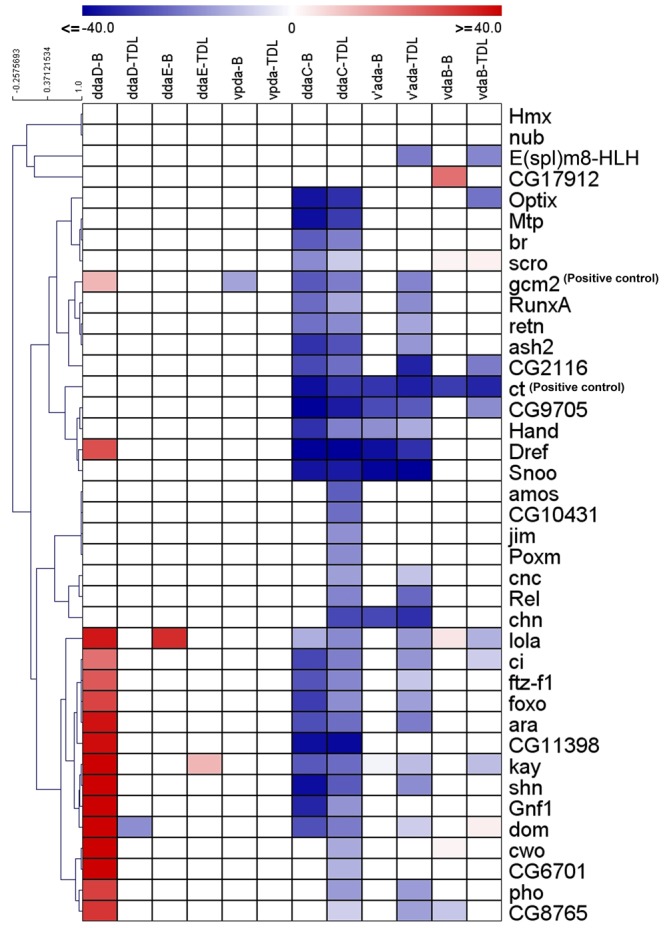
Hierarchical clustering representation of RNAi phenotypic screen data. Hierarchically clustered heat-maps of the quantified total dendritic length (TDL) and total dendritic branches (B) are represented by a color-code (red and blue), where red represent a significant increase in the phenotypic value and blue represents a significant decrease in phenotypic value according to the designated scale. All non-significant values were thresholded to zero (2 tailed Students t-test, p≤0.05). Here the quantified neurometric parameters from each experiment have been converted to a percentage change from control for normalization and to reflect both positive and negative changes from the controls when compared to the experimentals.

In order to create an unbiased platform for statistical characterization of dendritic phenotypes emerging from our functional validation screen, we developed and optimized a strategy for semi-automated quantification of dendritic arbors that dramatically improves throughput of neuronal reconstructions (see Materials and Methods). For the current study, over 4,900 neuronal images of knockdown phenotypes from all C-I and C-IV subtypes were collected and recorded for neurometric analyses. Key morphological features, including total dendritic length and total dendritic branches, were quantified for statistically significant changes relative to controls. Moreover, neurometric quantitative analyses were used as criteria for positive RNAi hit selection based upon the dendritic phenotypes observed in our qualitative screen. Collectively, these quantitative analyses revealed that ∼95% of the TFs screened (35 of 37) resulted in a phenotype in C-IV neurons and ∼49% resulted in RNAi-induced phenotypes in C-I neurons (18 of 37) that were significantly different than controls in at least 2 independent *UAS-IR* constructs (Figs. 4–7, Table S6).

### Transcriptional regulation of class and subtype specific dendritogenesis

C-I neurons are the least morphologically complex among the *Drosophila* da neurons innervating the larval cuticle, and consist of three neurons, ddaD, ddaE and vpda [7], hereafter referred to as C-I subtypes. Each enriched or differentially expressed TF was phenotypically screened using multiple *UAS-IR* lines and total dendritic branches and total dendritic length was quantified for each condition. A total of 18 of the 37 TFs examined (∼49%) resulted in statistically significant changes in total dendritic length and/or total dendritic branches in at least 2 independent *UAS-IR* lines among C-I neuron subtypes (Fig. 4). Interestingly, a larger number of transcription factors resulted in dendritic phenotypes in ddaD (18) versus ddaE (2) or vpda (1) C-I neurons (Fig. 4G). These also included several TFs that resulted in dendritic phenotypes in select C-I subtypes. For example, RNAi of the Zinc-finger BED-type domain containing TF *DNA replication-related element factor* (*DREF*) resulted in an overall increase in total dendritic branches in ddaD, but not in ddaE or vpda (Fig. 4A–F). Similarly, knocking down *kayak* (*kay*) (Fig. 4O) or *longitudinals lacking* (*lola*) (Fig. 4P) resulted in dendritic phenotypes only in ddaD and ddaE neurons, but not in vpda (Fig. 4A–F). To determine whether the observed subtype-specific effects may be due to variable expressivity of the C-I *GAL4* driver used, we quantitatively analyzed the average GFP fluorescent intensity values normalized to the area of the cell body for each of three C-I neurons. These results revealed relatively equivalent expression levels for ddaE and vpda and lower GFP expression levels in ddaD (∼45% lower) (Fig. S2A). The fact that we observe a higher frequency of subtype specific TF-induced phenotypic defects in ddaD relative to ddaE or vpda (Fig. 4G) argues against these phenotypic differences being attributable to heterogeneous *GAL4* expression levels in C-I neurons.

In contrast to C-I neurons, dendrites of C-IV da neurons elaborate extensively and display a space filling dendritic architecture with respect to covering their receptive field [7], [46]. Moreover, these neurons provide a complete, non-redundant coverage of the body wall of *Drosophila* larvae, exhibit dendritic tiling, and have been implicated in a range of sensory modalities [47]–[50]. Using the same phenotypic screening and hit-selection parameters applied to C-I neurons, 35 of the 37 TFs (∼95%) tested in C-IV neurons resulted in robust and consistent dendritic defects in at least 2 independent *UAS-IR* lines (Figs. 5–6). As observed with C-I neurons, each class IV subtype (ddaC, v’ada, vdaB) exhibited varying phenotypic effects on dendrite development with disruption of distinct TFs. Among the three C-IV subtypes, the maximum number of TFs exhibited phenotypes in ddaC neurons (34), followed by v’ada (19) and vdaB (12). Only 9 of the 35 TFs phenotypically validated in C-IV neurons resulted in dendritic defects in all three subtypes (ddaC, v’ada, vdaB) (Fig. 6). For example, RNAi of the three previously uncharacterized TFs *CG2116, CG8765 and CG9705* all resulted in an overall reduction in total dendritic length and total dendritic branching in all three C-IV subtypes (Fig. 5C, Fig. 6). On the other hand, knocking down some TFs uniquely affected only a subset of C-IV neurons (Fig. 6). RNAi of the following (5) Zinc-finger, C2H2-type domain containing TFs *CG10431*, *jim*, *CG11398*, *CG6701* and *broad* (*br*) all resulted in moderate to strong reduction in dendritic complexity exclusively in ddaC neurons (Fig. 6G). As with the C-I analyses, we examined whether the observed subtype-specific phenotypic defects could be potentially due to heterogeneous *GAL4* expression. These analyses revealed relatively even expression levels for ddaC and vdaB relative to the somewhat higher expression levels in v’ada (∼20% higher) (Fig. S2B). Despite these variations in *GAL4* expression levels across C-IV subtypes, we observed several TFs that resulted in phenotypic defects in only ddaC neurons (Fig. 6G) which, again, argues against these subtype-specific phenotypes being due to heterogeneous *GAL4* expression levels in C-IV neurons.

Interestingly, selected TFs exhibited context-dependent and opposing dendritic defects upon knockdown in different C-IV subtypes. For example, RNAi-mediated disruption of the helix-loop-helix domain containing TF *clockwork orange* (*cwo*) resulted in a moderate reduction in complexity among ddaC and v’ada neurons (Fig. 5D,I), but caused an increase in dendritic complexity in the ventral vdaB neurons (Fig. 5N, Fig. 6). This example provides a glimpse into the complex context-dependent regulatory roles TFs play in regulating both class-specific and subtype-specific dendrite development.

### Hierarchical clustering of quantified phenotypic screen results

Hierarchical clustering analysis offers a powerful way to predict functionally similar transcription factors, based on the resulting phenotypes generated. As such, quantified image data was normalized against controls (*C-I/ C-IV-GAL4* lines crossed to *Oregon-R*) and used to generate hierarchical cluster maps from the quantified image features and non-significant values were thresholded to zero (2 tailed Students t-test, p<0.05) (Fig. 7, Table S7). The quantified values of total dendritic length and branch number are represented as the percent change from controls and are denoted by a color-code where red values indicate an increase in the phenotypic metric and blue represents a decrease in phenotypic metric according to the designated scale (Fig. 7).

To systematically study and compare the role of TFs in governing both da neuron subclass and subtype specific differences, we analyzed the phenotypes exhibited by each individual subtype for both C-I (ddaD, ddaE and vpda) and C-IV neurons (ddaC, v’ada, vdaB). Interestingly, only a relatively small percentage of TFs exhibited uniform phenotypes in all subtypes for a given da neuron subclass. For example, only about 30% of TFs functionally validated in C-IV neurons caused uniform phenotypes in all three subtypes (ddaC, v’ada, vdaB) (Fig. 6G, Fig. 7). This was seen even more dramatically in C-I neurons, where none of the 18 functionally validated TFs exhibited uniform phenotypes within the three C-I subtypes (ddaD, ddaE, vpda) (Fig. 4G, Fig. 7).

### C-IV, but not C-I, dendrites exhibit scaling properties

**Figure 8:**
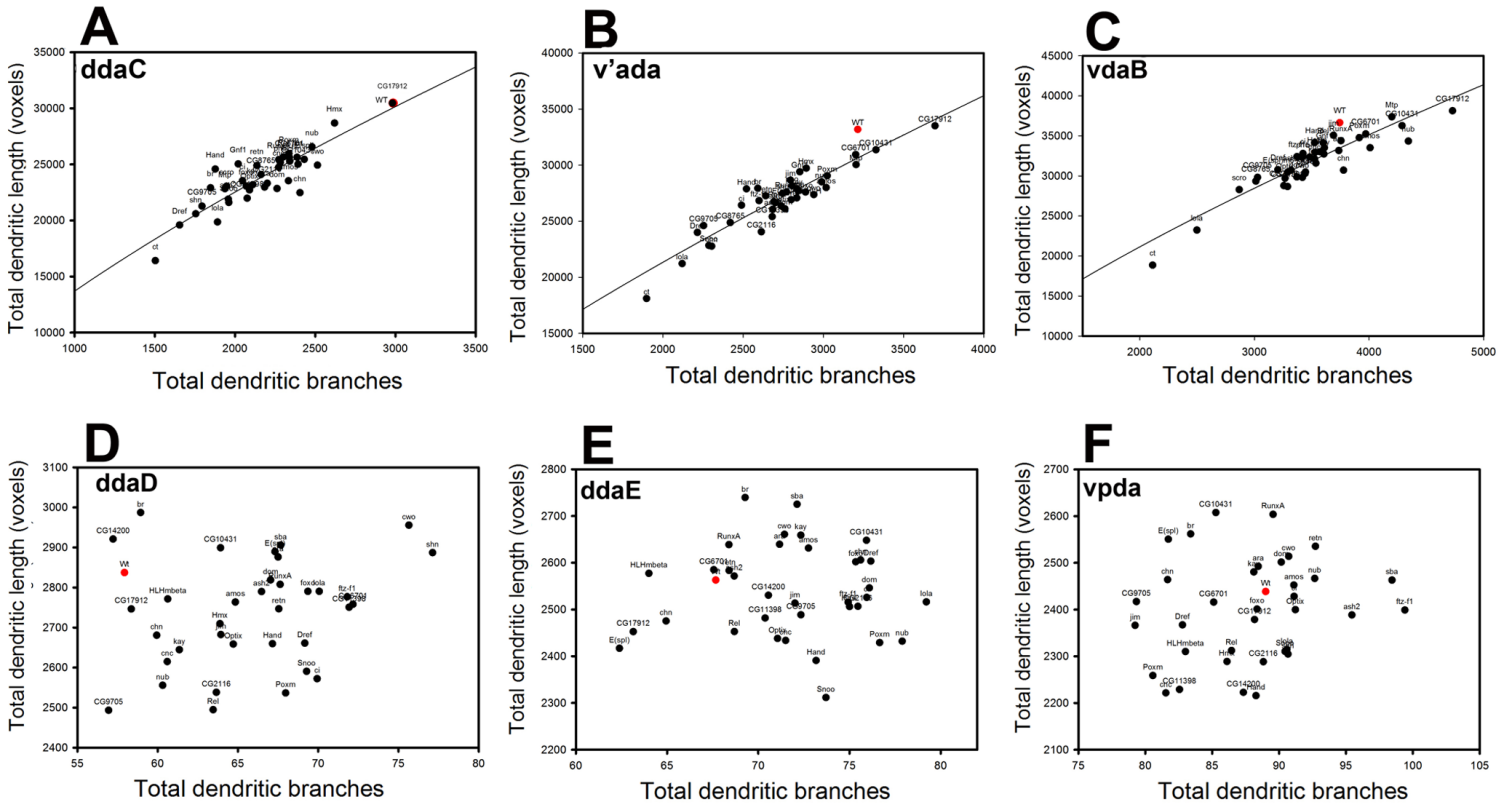
C-IV, but not C-I, neurons exhibit dendritic scaling properties. Dendritic scaling relationship between the total dendritic branches and total dendritic length for C-IV (**A-C**) and C-I (**D-F**) are represented as a 2-dimensional scatterplot for both wild-type (red) and the functionally validated enriched TFs (black). The relationships are constant between the two parameters for a wide range of values for C-IV neurons (**A-C**), as represented by the line of best-fit described by the equation *L =  a.B^x^*, where *L* is total dendritic length, *B* is total dendritic branches and *a* is an arbitrary constant. The average power value (*x*) was found to ∼0.73 (± 0.01 s.d.). No clear relationship was detected between the *L* and *B* parameters for C-I neurons (**D-F**).

Morphologically, C-I and C-IV neurons exhibit dramatically different receptive field innervations with C-I neurons displaying selective coverage and C-IV neurons displaying space-filling properties [4]–[6]. A recent study has demonstrated that a wide variety of neurons follow scaling laws to establish optimal dendritic branching and predicts a 2/3 power law relationship between total dendritic length and number of dendritic branch points [51]. In light of the large number of C-I and C-IV dendritic reconstructions performed in our screen, we were interested in the potential that this 2/3 power law could be used to account for the dendritic arborization profiles observed in C-I and/or C-IV da neurons and how post-mitotic TF knockdown might impact potential scaling properties of dendrites. When we plot the total dendritic length of each neuronal subclass with respect to total branches, we see that the trend of C-IV phenotypic distribution clusters tightly around the line of best fit with the equation *L =  a.B^x^*, where *L* is total dendritic length, *B* is total dendritic branches, a is an arbitrary constant. We find that the average power value (*x*) is 0.73 (+/– 0.01 s.d.) (Fig. 8) which largely agrees with the 2/3 power law previously reported [51]. In stark contrast, there was no discernible relationship between total dendritic branches and total dendritic length of C-I neurons (Fig. 8). This observation potentially highlights a fundamental difference between these two neuronal subclasses. Our finding is supported by previous studies that show that C-IV neurons completely and non-redundantly fill dendritic territories using dendritic self-avoidance as a key self-organizing principle, while in contrast, C-I neurons use an independent mechanism to achieve selective innervation of specific dendritic territories [4]-[6]. Moreover, our findings suggest that C-IV neurons exhibit these dendritic scaling properties largely independent of enriched or differentially expressed TF activity.

## Discussion

We have used an unbiased, functional genomic approach coupled with a systematic RNAi screen to identify molecular mechanisms underlying class-specific dendrite development of the *Drosophila* da neurons. Through our microarray analyses, we have identified genes that are differentially expressed within C-I and C-IV neurons, in addition to those that are commonly enriched among these two classes. This dataset identified a large number of genes belonging to the category of signalling molecules, ion channels, receptors, enzymes, cytoskeletal regulators and transcription factors, as well as those with no known function (Table S1–S4). From this dataset, we have validated functional roles of 37 TFs, of which 18 are required for C-I dendritogenesis and 35 function in regulating C-IV dendrite development.

### Transcriptional profiling as a predictive tool to investigate class-specific dendrite development

Guided by the microarray data, our TF screen resulted a larger number of positive hits than may otherwise have been expected. For example, ∼95% of the TFs examined in C-IVs resulted in strong and reproducible dendritic defects using independent RNAi constructs. Similarly, ∼49% of the 37 TFs investigated resulted in dendritic phenotypes in C-I neurons. For our analysis, since the neuronal samples are compared to whole larva lysate RNA, inherently this approach is aimed to identify only genes significantly enriched over background. Hence, not surprisingly, several TFs previously implicated in mediating da neuron dendrite morphogenesis including Knot, Cut, Spineless and Abrupt [4] were not uncovered due to their high expression in several non-da neuron tissue tissues including high expression in the ventral nerve chord [29]. We also observed that, the relative microarray TF expression levels seem, in some cases, to be only weakly correlated with the observed phenotypic probability and strength (Table S10). For example, disruptions in the TF *araucan* (*ara*), which is differentially upregulated in C-IV neurons (Fig. 3C), also resulted in a significant dendritic phenotype in C-I neurons (Fig. 4), despite the fact that our microarray studies demonstrate *ara* to be downregulated by nearly 2 fold in C-I neurons compared to whole larval controls. This is not surprising because, even though *ara* is detected to be robustly expressed, it may still be downregulated in C-I neurons in comparison to whole larvae RNA samples. Hence, while differential expression analysis is an excellent method to prioritize a gene-list for analysis, the biological interpretation must be made with caution.

### Role of protein biosynthesis and proteolysis in dendrite development

Protein synthesis and proteolysis are complimentary mechanisms that are extremely critical for neural development and plasticity [52]. One of the most interesting results of our comparative microarray analyses is the differential regulation of genes involved in protein biosynthesis and proteolysis between C-I and C-IV neurons. For example, ribosomal genes were the most statistically enriched set among genes upregulated uniquely in C-IV neurons (p = 4.5×10^−14^), while genes involved in proteolysis were the most significantly downregulated (p = 9.11×10^−6^) (Fig. 2, Table S1-S4). In contrast to this, genes involved in proteolysis were highly enriched among genes uniquely upregulated in C-I neurons (p = 3.3×10^−4^) (Fig. 2, Table S1-S4). This might indicate that not only are C-IV neurons negatively proteolytic, but are also actively anabolic, while on the other hand, C-I neurons may be actively catabolic. Proteolysis plays a critical role in regulating a great variety of cellular processes such as activating or deactivating enzymes, receptors and transcription factors among other molecules [52]–[54]. However, how the proteolytic pathway specifically intersects with transcriptional programs to regulate class-specific dendrite morphogenesis remains to be understood. Conversely, it would also be interesting to understand if transcriptional programs might mediate dendritic diversity via differential regulation of proteolytic pathways.

### Role of mitochondria in class-specific dendrite development

Mitochondria play a core role in neuronal function by both powering the cell via oxidative phosphorylation and maintaining cellular homeostasis [55]. Our microarray results indicates that several genes associated with oxidative phosphorylation and mitochondria exhibit the most differential expression levels between C-I and C-IV neurons (Fig. 2, Table S1-S4). These results might reflect the underlying differences in energy demands between C-I and C-IV neurons. This hypothesis is supported by a recent study which demonstrated that the mitochondrial protein Preli-like is required for the maintenance of mitochondrial integrity and is critical for proper growth and maintenance of C-IV da neurons [56]. Moreover, it is quite interesting to note that while disrupting mitochondrial function resulted in simplified C-IV dendritic arbors, it did not have any significant effect on C-I, C-II or C-III da neurons [56]. Taken together, these observations suggest that C-I and C-IV neurons may differ dramatically in their metabolic demands. This raises an interesting possibility of whether downregulation of mitochondrial function might be a potential mechanism used to restrict the growth of dendritic arbors to generate a simplified architecture.

While TFs clearly play important roles in directing class-specific dendritic patterning, the downstream targets of these TFs that are involved in the process of dendrite morphogenesis remain poorly understood. A recent study has identified the localization of nuclear TFs in mammalian mitochondria [57], raising the exciting potential of direct mitochondrial gene expression control by nuclear TFs. Whether class-specific dendritic patterning might be affected in part via the direct transcriptional regulation of mitochondrial genes by nuclear TFs remains to be explored.

### Post-mitotic and context-dependent roles of TFs in class-specific dendritogenesis

The view that different TFs are dedicated to distinct phases of neuronal morphogenesis has proven to be an oversimplification. One of the important conclusions of recent studies is that TFs continue to play important roles in post-mitotic neurons with respect to mediating distinct aspects of development [4], [58]. In a relatively recent genome-wide screen for transcriptional regulators of C-I dendrite development, 78 TFs were identified [9]. While a direct comparison between that study [9] and the current study is not possible due to significant differences in experimental design, it is interesting to note that there was very little overlap between the TFs identified in the two studies. For example, the previous screen was conducted via injection of double-stranded RNAi into syncytial blastoderm embryos [9], while we have performed our knockdown using class-specific *GAL4* drivers that start driving expression in da neurons 14–17 hours post-egg lay, at which point the da neurons begin elaborating their dendrites. Hence, the TFs identified in our screen likely play important roles post-mitotically to regulate dendritogenesis. This might be one of the reasons why we did not observe any TFs that resulted in changes in cell numbers. Moreover, our study focused on those TFs that were significantly enriched as well as differentially expressed in C-I and C-IV da neurons, whereas the previous study examined all TFs independent of any comparative expression data. Taken together, the results of our study along with those from the previous screen in C-I neurons [9] suggest that a large number of TFs are required continually post-mitosis for development and maintenance of class-specific dendritic architectures. Our microarray data further reveals that almost twice the number of TFs are uniquely enriched in C-IV neurons (17) versus C-I neurons (9). Consistent with this observation, our candidate RNAi screen of 37 TF regulators also resulted in phenotypes for 18 TFs in C-I neurons and 35 TFs in C-IV neurons. These observations suggest that C-IV neurons may require a larger and more diverse repertoire of TFs when compared to C-I neurons and that a significantly larger number of TFs might be essential for regulating the dendritic patterning of more complex dendritic architectures.

One of the most interesting findings from our analyses was the observation that individual TFs exhibited distinct phenotypic effects to RNAi knockdown in only a subset of neurons within a given da neuron subclass (*e.g.* C-I or C-IV) and that these subtype-specific effects are not due simply to variable expressivity of the *GAL4* drivers within the individual da neuron subtypes (Fig. S2). While C-I and C-IV neurons share clear similarities in their dendritic growth and branching patterns that allow for sub-classification [7], they also exhibit a variety of morphological differences with respect to axon orientation in context to dendrites and body axes, number of primary and secondary branches, shape of the soma and dendritic routing to territories these neurons cover along the larval body wall. Our findings revealed that only ∼30% of the TFs that were functionally validated produced uniform phenotypic effects in all three C-IV subtypes, whereas none of the functionally validated TFs exhibited consistent phenotypic effects in C-I subtypes. Consistent with our findings, previous studies have revealed similar results where disruptions in *Cubitus interruptus* specifically effect only dorsal C-I neurons without affecting the ventral vpda neuron [9] and null mutations in *cut* have been shown to exhibit neuron-specific effects among class III da neurons [22]. These findings reveal that molecular heterogeneity likely plays a critical role in the development of subtype-specific dendritic morphologies. In addition to intrinsic, cell-autonomous effects of TFs on subtype-specific dendrite morphogenesis, da neuron also likely rely upon extrinsic positional cues for proper patterning. Previous studies have demonstrated that signalling from adjacent cells can regulate dendrite development in a non cell-autonomous manner [59]. As embryonic patterning is highly dependent upon differential morphogenetic gradients across both the anterior-posterior and dorsal-ventral axes, it is logical to speculate that in order to respond to these developmental cues each neuron across these axes might modulate the repertoire of signalling molecules to which they can respond via TF-initiated changes in gene expression. Subtype-specific TF function may therefore modulate differential gene expression that may manifest as distinctive dendritic growth and branching patterns appropriate to the local environment of the neuron’s receptive field. In light of this, the phenotypic differences observed among subtypes of the same da neuron subclass may be a reflection of the underlying molecular heterogeneity that drives subtype-specific dendrite arborization patterning.

Our results further reveal that TFs are highly context-dependent in regulating class and subtype specific dendrite development. We observed that TFs not only work in a context-dependent manner between two independent neuron subclasses, but also function between distinct neuronal subclasses to produce opposing results. This can be clearly seen in the following examples where RNAi of *Dref, cwo, kay* and *lola* led to a decreased dendritic complexity in C-IV neurons, while promoting dendritic arborization of C-I neurons (Fig. 7). Moreover, we observed that disruptions in a large number of TFs resulted in phenotypes only in selected subtypes of a given neuronal class (Figs. 4–7, Table S6). Taken together, these findings indicate that dendritogenesis in neuronal subclasses, and even distinct subtypes within a given subclass (*e.g.* ddaC among C-IV neurons or ddaD among C-I neurons), can be molecularly very distinct.

Collectively, the findings from our comparative transcriptional profiling, bioinformatic analyses, functional RNAi screen and neurometric quantitative studies provide a global framework of the roles transcriptional regulation plays in directing class and subtype-specific dendrite development. In addition, given that hierarchical clustering reveals groups of transcription factors that appear to contribute predominantly to class-specific dendrite development future studies will be required to investigate the potential of combinatorial modes of action among these transcription factors. Interestingly, we find that ∼78% of the TFs implicated in C-I dendritogenesis (14 of 18) and ∼66% of TFs regulating C-IV development (23 of 35) are evolutionarily conserved in humans, with many implicated in nervous system disease (Tables S8, S9). Thus, our work may also potentially shed new light for future studies of homologous gene function in the vertebrate nervous system. Finally, as da neurons have become a widely employed model system for investigating both the regulation of dendrite morphogenesis [4]–[6] and somatosensory behavior [50], our report of C-I and C-IV specific gene expression profiles should prove a highly valuable resource for circumventing issues associated with genetic pleiotropy and thereby facilitating directed studies aimed at the dissection of the putative molecular programs underlying class-specific dendrite morphogenesis and sensory neuron function.

## Materials and Methods

### Fly strains and genetics

*Drosophila* stocks were raised on standard cornmeal-molasses-agar media at 25°C. Fly strains used in these studies were obtained from Bloomington (*GAL4^477^*,*UAS-mCD8::GFP*, *DTS(1)/CyO,tubP-GAL80*, *UAS-RNAi* TRiP lines (JF lines)), Vienna *Drosophila* RNAi Center (*UAS-RNAi* GD and KK lines) and other sources (*GAL4^ppk.1.9^,UASmCD8::GFP, UAS-cut*) [10], [21], [22], *ppk-GAL80* [24], and *GAL4^221^*,*UAS-mCD8::GFP* [22]. *Oregon-R* was used as a wild-type strain. For each TF, a minimum of (2) and up to (5) gene-specific *UAS-RNAi* lines were carefully selected against off-target effects wherever possible and crosses were performed at 29°C (Table S11).

### RNAi phenotypic screening and live image confocal microscopy

Virgin flies from individual *GAL4* lines of the genotype *GAL4^477^*,*UAS-mCD8::GFP*/*CyO,tubP-GAL80*;*GAL4^ppk.1.9^*,*UAS-mCD8::GFP* (C-IV GAL4), *GAL80^ppk1.9^, GAL4^221^*, *UAS-mCD8::GFP* (C-I GAL4) were collected and aged for 2 days prior to crossing them to individual, gene-specific *UAS-RNAi* males (Table S11) or crossed to wild-type *Oregon-R* males as control, followed by rearing at 29°C. The *UAS-RNAi* lines were each assigned a randomly generated numerical code and screening was conducted double-blind to the identity of the gene targeted by the *UAS-RNAi* construct. 6–10 fluorescent third instar larvae bearing both the *GAL4* and *UAS-RNAi* were analyzed via live image confocal microscopy and representative image data was collected. For live confocal analyses, larvae were placed on a microscope slide, immersed in 1:5 (v/v) diethyl ether to halocarbon oil and covered with a 22×50 mm glass coverslip. Neurons expressing GFP were visualized on a Nikon C1 Plus confocal microscope. Images were collected as z-stacks using a 20X oil immersion lens at a step-size of 2.5 µm and 1024×1024 resolution. Quantitative analyses of C-I and C-IV *GAL4* expression levels were performed as previously described [19], [20]. Briefly, third instar larvae bearing either the C-I or C-IV reporter strains used in the RNAi phenotypic screen were subjected to live-image confocal microscopy and the average GFP fluorescent intensity was quantified at the level of the cell body using equivalent confocal laser power and gain settings for each *GAL4* reporter strain. Images were imported into Photoshop (Adobe) for measurements of integrated pixel density and the average fluorescent intensity expression values were normalized to the area of the cell body for each C-I and C-IV da neuron subtype.

### Cell isolation, purification and qRT-PCR

The isolation and purification of C-I and C-IV da neurons was performed as previously described [17]. Briefly, 40-50 age-matched third instar larvae expressing *mCD8::GFP* under the control of the either *GAL4^ppk.1.9^*, or *GAL80^ppk.1.9^,GAL4^221^* drivers were collected and washed several times in ddH20. The larvae were then rinsed in RNAse away, ddH20 and finally dissected. The tissue was then dissociated using a combination of enzymatic and mechanical perturbations to yield single cell suspensions which were filtered using a 30µm membrane. The filtrate is then incubated with superparamagnetic beads (Dynabeads MyOne Streptavidin T1, Invitrogen) coupled with biotinylated mouse anti-CD8a antibody (eBioscience) for 60 minutes. Finally the da neurons attached to the magnetic beads were then separated using a powerful magnetic field. The isolated neurons were washed at least five times with PBS to remove any potential non-specific cells and the quality and purity of isolated neurons was assessed under a stereo-fluorescent microscope equipped with phase contrast for examining the number of fluorescent (GFP-positive) vs. non-fluorescent (GFP-negative) cells. Only if the isolated cells were free of cellular debris and non-specific (*i.e.* non-fluorescing) contaminants were they retained. The purified C-I and C-IV neuron populations were then lysed in SuperAmp™ (Miltenyi Biotec) RNA lysis buffer followed by storage at -80°C. For whole larvae controls, 10 third instar larvae were homogenized in PicoPure RNA isolation buffer (Invitrogen), following which RNA was eluted as per manufacturer protocols. In order to process the whole larvae RNA identically to the da neuron samples, 10 microliters of whole-larvae RNA, diluted to the same levels as da neuron samples, was added to SuperAmp SuperAmp™ (Miltenyi Biotec) buffer and stored at -80°C. For qRT-PCR analyses (see Fig. S1), RNA was extracted from isolated C-I or C-IV da neurons (in the presence or absence of a *UAS-cut* transgene) as an independent measure of cell purity. These analyses were performed as previously described [19], [20] using the following pre-validated Qiagen QuantiTect Primer Assays (Qiagen, Germantown, MD, USA): *cut* (QT00501389) and expression data was normalized using primers for *GAPDH2* (QT00922957) and *RpL32* (QT00985677).

### Microarray analyses

mRNA isolation, amplification, labelling, hybridization, and microarray analyses were conducted by Miltenyi Biotec. Briefly, mRNA was isolated from independent pools of da neurons (1000–1500 neurons/pool) via magnetic bead technology and the sample quality verified on an Agilent Bioanalyzer 2100 prior to amplification. Same quantity of whole larval RNA lysate was processed as the control sample. SuperAmp RNA amplification was performed on all the samples according to Miltenyi Biotec’s global PCR protocol using mRNA-derived cDNA. Amplified cDNA samples were quantified using the ND-1000 Spectrophotometer (NanoDropTechnologies) and the sample integrity verified via Agilent 2100 Bioanalyzer analyses (Agilent Technologies). 250 ng of each of the cDNAs were used as template for Cy3 labeling followed by hybridization to Agilent whole *Drosophila melanogaster* genome oligo microarrays (4×44 K). The Agilent Feature Extraction Software (FES) was used to read out and process the replicate microarray image files. The software was used to determine feature intensities and perform background subtraction, reject outliers and calculate statistical confidences. For determination of gene expression above background FES derived output data files were further analyzed using the Rosetta Resolver gene expression data analysis system (Rosetta Biosoftware). For differential gene expression analysis, raw microarray data was normalized and analyzed by GeneSpring GX 12.0 software (Agilent). Briefly, raw data was normalized using quantile-normalization algorithm and all raw signals were thresholded to 1 and data was baseline transformed to the median signal intensity value of all arrays. Probes that were either saturated, non-uniform or population outliers were flagged as “Compromised’ and were filtered out. Further, only spots whose mean signal intensity was significantly greater than the background (as established by a 2-sided t-test, p<0.01) were considered to be expressed above background. Arrays were grouped (C-I, C-IV or Oregon R) and signal values were averaged over replicates for the analyses. To identify differentially enriched genes that have significantly different expression levels across C-I and C-IV neurons when compared to whole larvae, a one-way ANOVA test with asymptotic P-Value computation and Benjamini-Hochberg multiple testing correction along with Tukey Honestly Significant Difference (HSD) test was performed on the array data against pairs of conditions (C-I vs. whole-larvae, C-IV vs. whole larvae). A stringent corrected P-Value cut-off of 0.01 was applied to filter the results. Finally, fold change analysis was performed on the statistically analyzed datasets to identify genes differentially expressed over 2-fold when compared to whole larvae controls. Microarray data, including metadata, raw data, and normalized datasets, has been deposited into the Gene Expression Omnibus (GEO) under the Accession Number GSE46154.

### Neurometric quantification and Hit selection

Raw confocal images were manually curated to eliminate non-specific auto-fluorescent spots such as the larval denticle belts. The raw pixel intensity for each image was globally thresholded and converted to a binary file format in Photoshop™(Adobe). Background image noise was filtered out using the Analyze Particles plugin (http://rsbweb.nih.gov/ij/docs/menus/analyze.html#ap) in ImageJ (Size (pixels^2^) ≤50 microns, Circularity ≥0.35) [60]. Next, images were skeletonized (conversion to 1 pixel wide “skeletons”) using the Skeletonize3D plugin (http://fiji.sc/wiki/index.php/Skeletonize3D) in Fiji/ImageJ followed by use of the Analyze Skeleton Fiji/ImageJ plugin (http://fiji.sc/wiki/index.php/AnalyzeSkeleton) for the output of quantitative neurometric measures of dendritic morphology [61], [62]. Images with low fluorescence or high background were eliminated from analysis. Quantitative neurometric information including total dendritic length and total dendritic branches was extracted and compiled using custom Python algorithms freely available upon request. The custom Python scripts were used to compile the output data from the Analyze Skeleton ImageJ plugin and the compiled output data was imported into Excel (Microsoft). Neurometric data was analyzed in Microsoft Excel 2010 (Microsoft) and statistical tests were performed (Student’s t-test) and plotted in SigmaPlot 11.0 (Systat Software). A transcription factor was deemed as a true hit only if at least two independent *UAS-RNAi* lines resulted in statistically significant changes from wild-type controls in a given phenotypic category (p≤0.05). Where multiple RNAi lines yielded mixed phenotypes, the TF was assigned the strongest and most consistent phenotype. The total number of *UAS-IR* lines included in this study were 103 (97 TF lines and 6 control lines for *ct* (3) and *gcm2* (3)). The number of C-I images reconstructed (n = 9 for each *UAS-IR* line per C-I subtype) was 2,163 and the number of C-IV images reconstructed (n = 7 for each *UAS-IR* line per C-IV subtype) was 2,781 for a grand total of 4,944 reconstructed neurons.

### Bioinformatic analyses

Functional enrichment analysis was performed using DAVID [30], [31] to identify statistically over-represented functional gene classes. The up/downregulated gene list from the microarray analysis was used as input and all genes represented in the microarray were used as background. To avoid over counting duplicated genes present on the microarray, DAVID uses the Fisher’s Exact statistics to retain significant results calculated after redundancy in original probe IDs are removed [31]. The EASE Score, a modified Fisher Exact P-value ranges from 0 to 1 where a value of 0 represents perfect enrichment. A P-value threshold of 0.01 was used as a cut-off for enrichment in the annotation categories. InterPro annotations were used for classifying transcription factors based on enriched functional categories [63], [64]. TF mining was performed using the list of putative, site-specific TF’s obtained based on FlyBase/Gene Ontology annotation or the DNA-binding domain (DBD) TF Database [65], [66] (Table S12). Microarray data for this set of gene and statistical tests were performed on this list as described earlier using GeneSpring GX 12.00 (Agilent). Hierarchical clustering analysis was performed using the TM4 software suite [67]. Corresponding human orthologs were mapped using the g:Orth tool [68] (Tables S8,S9).

## Supporting Information

Figure S1 **qRT-PCR analyses of Cut overexpression in C-I and C-IV da neurons reveals specificity of the class-specific cell isolation relative to controls.** qRT-PCR results (*n* = 4) reveal that Cut overexpression in C-I and C-IV neurons, via the same class-specific *GAL4* drivers used in the wild-type C-I (*ppk-GAL80;GAL4^221^,UAS-mCD8::GFP*) and C-IV (*GAL4^ppk1.9^,UAS-mCD8::GFP*) isolations, results in a significant upregulation of *cut* mRNA levels relative to class-specific control neurons that lack the *UAS-cut* transgene. Analyses reveal a 14±4 fold upregulation of *cut* in C-I neurons relative to controls and a 4.4±1.2 fold upregulation of *cut* in C-IV neurons relative to controls. Controls are indicated by the dashed red line and all data are normalized to *GAPDH2* and *RpL32* expression levels. Quantitative data is average ± S.D. and *p* values for the Student’s t-test comparing experimental to control are expressed as follows: (***) *p*≤0.001.
(TIF)

Figure S2 **Quantitative analyses of C-I and C-IV** ***GAL4*** **reporter expression levels.** Average fluorescence intensity values of C-I and C-IV *GAL4* reporter lines used in the phenotypic screen were measured by quantifying the GFP expression levels normalized to the area of the cell body for each da neuron subtype in third instar larvae. **(A)** Analyses of the C-I reporter, *ppk-GAL80;GAL4^221^,UAS-mCD8::GFP*, reveal relatively equivalent expression levels for ddaE and vpda neurons and an approximate 45% lower level of GFP expression in ddaD. **(B)** Analyses of the C-IV reporter, *GAL4^477^,UAS-mCD8::GFP;ppk-GAL4,UASmCD8::GFP*, reveal relatively equivalent expression levels for ddaC and vdaB neurons and somewhat higher GFP expression level (∼20%) in v’ada neurons. Data is presented as mean fluorescence intensity normalized to cell body area ± S.D. and the n value is represented on the bar graphs.
(TIF)

Table S1 **DAVID functional enrichment analysis of transcripts uniquely up/down regulated by 2-fold or greater (p≤0.01, ANOVA and Tukey’s HSD) in C-I neurons with respect to whole larvae controls.**
(XLS)

Table S2 **DAVID functional enrichment analysis of transcripts uniquely up/down regulated by 2-fold or greater (p≤0.01, ANOVA and Tukey’s HSD) in C-IV neurons with respect to whole larvae controls.**
(XLS)

Table S3 **DAVID functional enrichment analysis of transcripts commonly up/down regulated by 2-fold or greater (p≤0.01, ANOVA and Tukey’s HSD) between C-I and C-IV neurons with respect to whole larvae controls.**
(XLS)

Table S4 **List of transcripts expressed inversely between C-I and C-IV neurons are shown (all transcripts that were both upregulated in one class of neuron (C-I/C-IV) by 2-fold or greater and were also downregulated in the other class (C-I/C-IV) by 2-fold or greater with respect to whole larval controls (p≤0.01, ANOVA and Tukey’s HSD).** Enriched gene categories obtained using DAVID analysis using inverse expression data as input are also provided.
(XLS)

Table S5 **List of 40 differentially expressed TFs identified via microarray analysis.** These TFs were upregulated either uniquely in C-I or C-IV, or were commonly upregulated in both C-I and C-IV neurons when compared to whole larvae controls (p≤0.01, ANOVA and Tukey’s HSD).
(XLS)

Table S6 **Summary of RNAi-induced phenotypic screening results (hit-list) of the 37 functionally validated TFs.** TFs that resulted in statistically significant dendritic phenotypes as compared to wild-type controls (p≤0.05, Student's t-test) in at least 2 independent *UAS-IR* transgenes in each of the C-I and C-IV da neuron subtypes are provided.
(XLS)

Table S7 **Quantitative RNAi screening parameters are presented for all the TFs screened via** ***in vivo*** **RNAi.** Phenotypic values for total dendritic length and total dendritic branches are shown as percentage change from wild-type for *UAS-IR* lines that resulted in statistically significant phenotypes (p<0.05, Student's t-test) in at least 2 independent transgenic RNAi constructs in each phenotypic category. These data were used to construct the hierarchical cluster presented in Fig. 7.
(XLS)

Table S8 **Human orthologs of the TFs identified and validated in C-I neurons (Table S6) are provided.**
(XLS)

Table S9 **Human orthologs of the TFs identified and validated in C-IV neurons (Table S6) are provided.**
(XLS)

Table S10 **Table presenting comparative data on microarray differential expression values and RNAi-induced phenotypic effects.** RNAi hits (from Table S6) are presented alongside of microarray differential expression values (Table S5).
(XLS)

Table S11 **List of** ***UAS-IR*** **transgenic stocks used in this study.**
(XLS)

Table S12 **TF mining of microarray data was performed using the list of putative, site-specific TF’s obtained based on FlyBase/Gene Ontology annotation or the DNA-binding domain (DBD) TF Database.**
(XLS)
