# Supplementary figures and images for: Functional Genomic Analyses of Two Morphologically Distinct Classes of Drosophila Sensory Neurons: Post-Mitotic Roles of Transcription Factors in Dendritic Patterning
Source: PLoS One. 2013 Aug 15;8(8):e72434. doi: 10.1371/journal.pone.0072434 (PMC3744488; doi:10.1371/journal.pone.0072434)

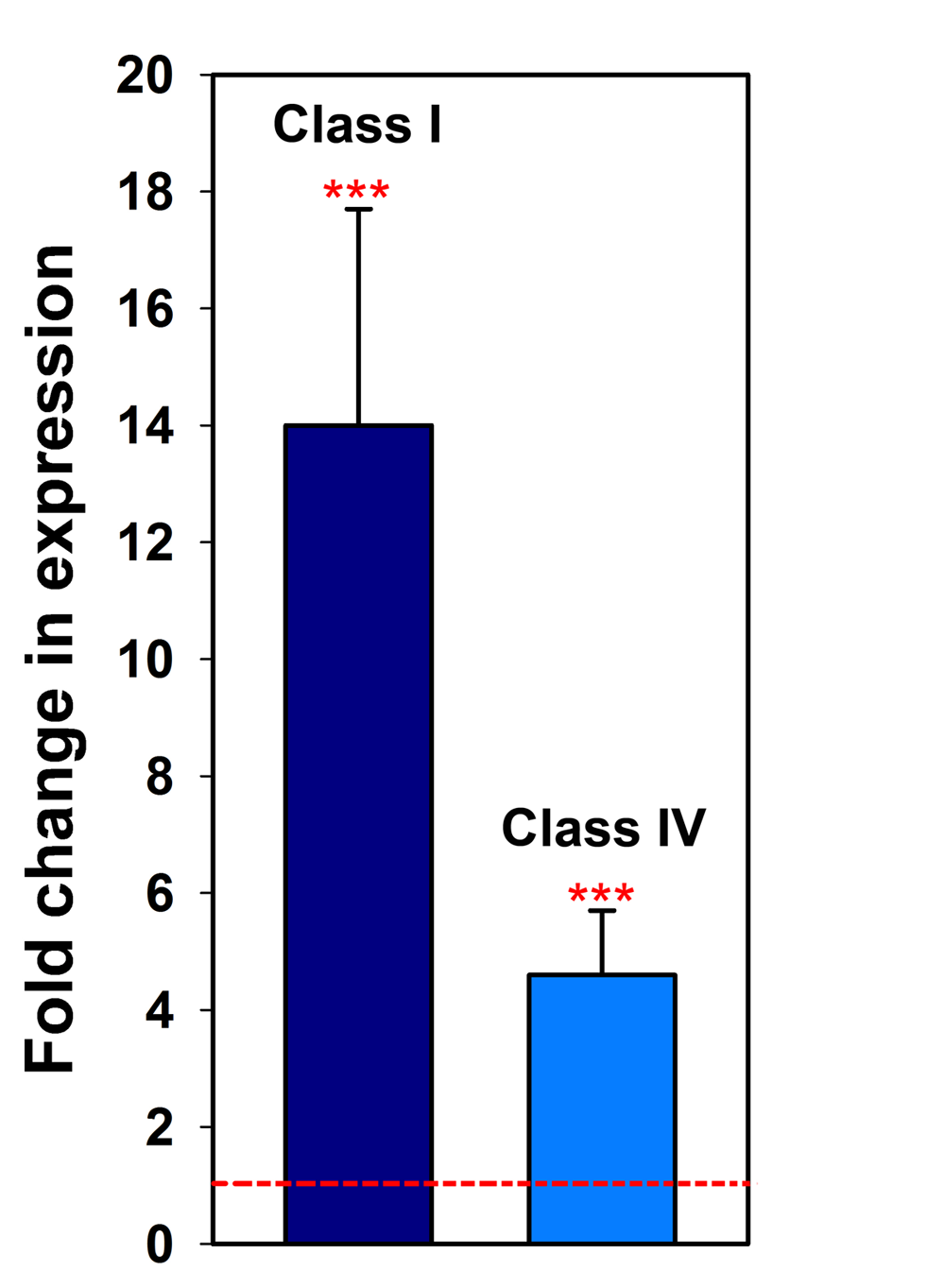

Supplement: Figure S1 — qRT-PCR analyses of Cut overexpression in C-I and C-IV da neurons reveals specificity of the class-specific cell isolation relative to controls. qRT-PCR results (n = 4) reveal that Cut overexpression in C-I and C-IV neurons, via the same class-specific GAL4 drivers used in the wild-type C-I (ppk-GAL80;GAL4221,UAS-mCD8::GFP) and C-IV (GAL4ppk1.9,UAS-mCD8::GFP) isolations, results in a significant upregulation of cut mRNA levels relative to class-specific control neurons that lack the UAS-cut transgene. Analyses reveal a 14±4 fold upregulation of cut in C-I neurons relative to controls and a 4.4±1.2 fold upregulation of cut in C-IV neurons relative to controls. Controls are indicated by the dashed red line and all data are normalized to GAPDH2 and RpL32 expression levels. Quantitative data is average ± S.D. and p values for the Student’s t-test comparing experimental to control are expressed as follows: (***) p≤0.001. (TIF) [file pone.0072434.s001.tif]

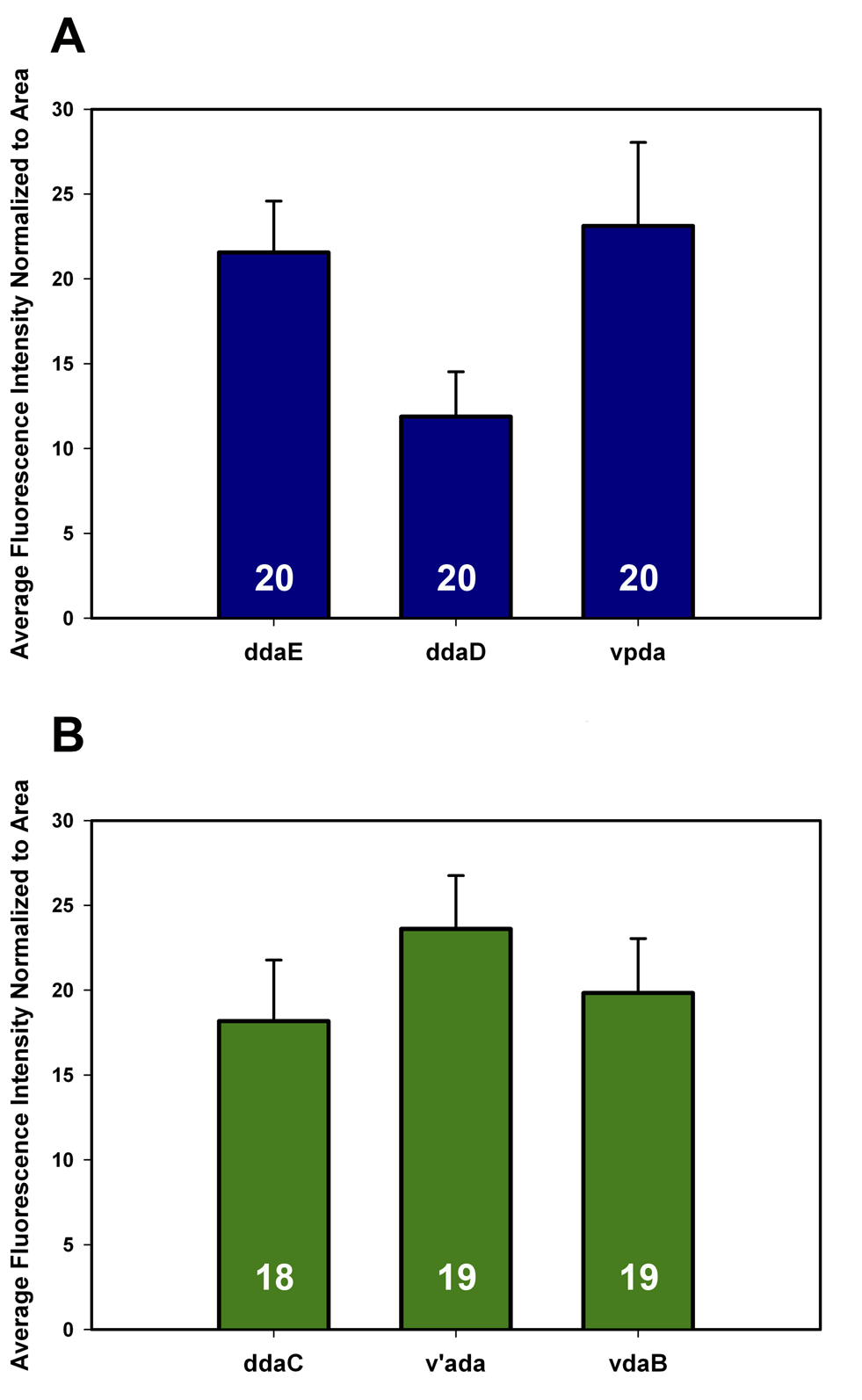

Supplement: Figure S2 — Quantitative analyses of C-I and C-IV GAL4 reporter expression levels. Average fluorescence intensity values of C-I and C-IV GAL4 reporter lines used in the phenotypic screen were measured by quantifying the GFP expression levels normalized to the area of the cell body for each da neuron subtype in third instar larvae. (A) Analyses of the C-I reporter, ppk-GAL80;GAL4221,UAS-mCD8::GFP, reveal relatively equivalent expression levels for ddaE and vpda neurons and an approximate 45% lower level of GFP expression in ddaD. (B) Analyses of the C-IV reporter, GAL4477,UAS-mCD8::GFP;ppk-GAL4,UASmCD8::GFP, reveal relatively equivalent expression levels for ddaC and vdaB neurons and somewhat higher GFP expression level (∼20%) in v’ada neurons. Data is presented as mean fluorescence intensity normalized to cell body area ± S.D. and the n value is represented on the bar graphs. (TIF) [file pone.0072434.s002.tif]
